# Effects of Adjuvants on HIV-1 Envelope Glycoprotein SOSIP Trimers *In Vitro*

**DOI:** 10.1128/JVI.00381-18

**Published:** 2018-06-13

**Authors:** Gabriel Ozorowski, Albert Cupo, Michael Golabek, Michelle LoPiccolo, Thomas A. Ketas, Matt Cavallary, Christopher A. Cottrell, P. J. Klasse, Andrew B. Ward, John P. Moore

**Affiliations:** a Department of Integrative Structural and Computational Biology, Center for HIV/AIDS Vaccine Immunology and Immunogen Discovery, International AIDS Vaccine Initiative Neutralizing Antibody Center, and Collaboration for AIDS Vaccine Discovery, The Scripps Research Institute, La Jolla, California, USA; b Department of Microbiology and Immunology, Weill Medical College of Cornell University, New York, New York, USA; Emory University

**Keywords:** HIV-1 vaccine, Env trimers, adjuvants

## Abstract

Native-like, soluble, recombinant SOSIP trimers of various designs and based on several *env* genes of human immunodeficiency virus type 1 (HIV-1) are being tested as immunogens in different animal models. These experiments almost always involve coformulating the trimers with an adjuvant to boost the magnitude of the immune responses. One factor relevant to the choice of an adjuvant is that it should not physically damage the immunogen or impede its ability to present relevant epitopes. As examples, an adjuvant formulation that includes harsh detergents could disrupt the structural integrity of a trimer, and any charged compounds in the formulation could bind to countercharged regions of the trimer and physically occlude nearby epitopes. While a few adjuvants have been tested for their potential effects on SOSIP trimers *in vitro*, there has been no systematic study. Here, we have assessed how nine different adjuvants of various compositions affect SOSIP trimers of the BG505 and B41 genotypes. We used negative-stain electron microscopy, thermal denaturation, and gel electrophoresis to evaluate effects on trimer integrity and immunoassays to measure effects on the presentation of various epitopes. We conclude that most of the tested adjuvants are benign from these perspectives, but some raise grounds for concern. An acidified alum formulation is highly disruptive to trimer integrity, and a DNA-based polyanionic CpG oligodeoxynucleotide adjuvant binds to trimers and occludes the trimer apex epitope for the PGT145 neutralizing antibody. The methods described here should be generalizable to protein subunit vaccines targeting various pathogens.

**IMPORTANCE** Adjuvant formulations increase the magnitude of immune responses to vaccine antigens. They are critically important for formulation of HIV-1 envelope glycoprotein (Env) vaccines intended to induce antibody production, as Env proteins are otherwise only very weakly immunogenic. The HIV-1 vaccine field now uses the well-defined structures of trimeric Env glycoproteins, like SOSIPs, to present multiple known epitopes for broad and potent neutralizing human antibodies in a native-like conformation. Successful adjuvant formulations must not disrupt how the trimers are folded, as that could adversely affect their performance as immunogens. We studied whether the various adjuvants most commonly used in animal experiments affect the integrity of two different SOSIP trimers *in vitro*. Most adjuvant classes are not problematic, but an aluminum sulfate formulation is highly damaging, as it exposes trimers to acidic pH and a nucleic acid-based adjuvant can bind to the trimer and block access to a key neutralizing epitope.

## INTRODUCTION

Various soluble envelope (Env) glycoproteins are being developed as vaccine candidates against human immunodeficiency virus type 1 (HIV-1) (1–5). The SOSIP design of trimers is now a widely used immunogen design platform (5–8). SOSIP trimers adopt a native-like conformation, in that they resemble the form of trimer that is found on infectious HIV-1 virions and present multiple epitopes for broadly neutralizing antibodies (bNAbs) (5–8). The latter property is relevant to the long-term goal of inducing this type of antibody via a suitably designed immunization regimen (7, 8). In practice, SOSIP trimers, like all other designs of HIV-1 Env protein, require delivery as an admixture with an adjuvant to boost their immunogenicity. It is important, however, that any chosen adjuvant does not adversely affect key properties of the immunogen. For example, harsh detergents or acidic buffers that are part of some adjuvant formulations could damage the structural integrity of the SOSIP trimer. Polyanion adjuvants such as nucleic acid mimics might bind to cationic regions and thereby directly or indirectly affect how the trimers display bNAb epitopes. Any such outcomes would compromise the rationale for engineering Env immunogens that present the most relevant epitopes to the immune system. By extension, the same concerns could apply to conformationally sensitive immunogens derived from other pathogens.

There have only been a few studies of how adjuvants interact with any HIV-1 Env proteins *in vitro* (9–12). For example, there is evidence that complete Freund's adjuvant impairs the conformational integrity of monomeric gp120, probably because its oil-based components disrupt stabilizing interactions within the hydrophobic core of the protein (11, 12). Polyanions, such as the RNA mimic poly(I·C), can inhibit HIV-1 infection *in vitro*, most likely via a charge-based interaction that compromises the natural functions of the virion-associated trimer (13–19). In principle, this type of association could occur with SOSIP trimer immunogens. ISCOMATRIX is an immunostimulating complex (ISCOM)-based adjuvant (20). This adjuvant has no visible effect on the conformation of the BG505 SOSIP.664 trimer, as judged by negative-stain electron microscopy (NS-EM) and reinforced by antigenicity studies (5). The lack of evidence for any adverse effects *in vitro* underpinned the use of ISCOMATRIX for initial rabbit and macaque immunogenicity studies (5, 7).

Here, we used a range of analytical techniques to investigate whether nine adjuvants of various compositions affect the integrity or antigenicity of the BG505 SOSIP.664 and B41 SOSIP.v4.1 trimers *in vitro*. We found that most adjuvant classes have negligible impact on SOSIP trimers, but formulations that require exposure to low pH (e.g., aluminum sulfate coprecipitation) should be avoided, as they cause dissociation of the trimer into monomers. The use of polyanionic oligodeoxynucleotide (ODN)-based adjuvants should be considered carefully, because we found that one representative of this class, CpG (ODN 1826), binds to the trimer apex and occludes access to the PGT145 bNAb epitope.

## RESULTS

### Choice of reagents.

Based in part on review of the relevant literature, we selected nine adjuvants to test for their effects on the conformation and/or antigenicity of clade A BG505 SOSIP.664 or clade B B41 SOSIP.v4.1 trimer (Tables 1, 2, and 3). The BG505 trimer has been produced under GMP conditions suitable for human clinical trials (21) and is widely used as a standard immunogen in animal models (5, 7, 22–24). The B41 trimer has also been tested in animals, and we considered that its lower thermal stability and apparent greater flexibility at the trimer apex than its BG505 counterpart might affect how it interacts with adjuvants (23, 25, 26). Both trimers were produced from stably transduced Chinese hamster ovary (CHO) cell lines to emulate our ongoing translational program (21). The chosen adjuvants included ones that have been used in animal studies involving SOSIP trimers or other gp140 proteins, as well as others that are plausible candidates for use in future SOSIP trimer clinical trials. These adjuvants included alum-, ISCOM-, or detergent-based formulations, small-molecule Toll-like receptor (TLR) activators, and nucleic acid-based polyanions (Tables 2 and 3). Because of their intrinsic properties and/or complex compositions, not every adjuvant could be studied in every assay. We describe below attempts to overcome various technical problems and obtain relevant data. First, we assessed whether each adjuvant class affected the structural integrity of the trimers. We then evaluated whether adjuvants impaired the presentation of various epitopes for bNAbs and, conversely, increased the exposure of some nonneutralizing antibody (non-NAb) epitopes.

**TABLE 1:** Summary of adjuvants used in immunization studies of selected soluble Env proteins

Reference	Animal species	Trimer	Adjuvant
Voss et al. (52)	Rabbit	Various SOSIP trimers, containing V2 mutations, based on clade B, AE, or AG sequences	ISCOMATRIX
Torrents de la Peña et al. (40)	Rabbit	Various SOSIP.v5	ISCOMATRIX
Capucci et al. (53)	Rabbit	BG505 SOSIP.664	ISCOMATRIX
Pauthner et al. (24)	Rhesus macaque	Various BG505 SOSIP designs	ISCOMATRIX
Ringe et al. (54)	Rabbit	V3-glycan mutants of BG505 or B41 SOSIP	ISCOMATRIX
Chuang et al. (21)	Guinea pig	BG505 DS-SOSIP	Adjuplex
Havenar-Daughton et al. (55)	Rhesus macaque	BG505 SOSIP.v5.2	MPL+R848 encapsulated PLGA nanoparticles, or ISCOMATRIX
Klasse et al. (23)	Rabbit	BG505, B41, CZA97, or DU422 SOSIP.664	ISCOMATRIX
Feng et al. (9)	Guinea pig	Various SOSIP or NFL designs, with or without cross-linking	ISCOMATRIX
Cheng et al. (56)	Guinea pig	BG505 SOSIP.664 alone or in complex with PGT145 Fab	Poly(I·C)
Sliepen et al. (57)	Mouse^*a*^ rabbit	BG505 SOSIP.664, as an individual trimer or as part of a ferritin-based nanoparticle	MPLA (mouse) or ISCOMATRIX (rabbit)
Hu et al. (58)	Mouse^*a*^	BG505 SOSIP.664	Various combinations of 1 or more of ISCOMATRIX, Abisco-100, Addavax, Asialo GM1, Adjuplex, complete or incomplete Freund's adjuvant, MPL, R848, CpG (ODN1826), IL-6, 7DW8-5, PLGA
Sanders et al. (5)	Rabbit	BG505 or B41 SOSIP.664	ISCOMATRIX

a Only studies using wild type mice are included, and not ones involving genetically altered mice (e.g., with knocked-in genes for human germ line B-cell precursors).

**TABLE 2:** Summary of biophysical analyses of how adjuvants affect trimer stability

Name	Composition	Category	Supplier	NanoDSF^*b*^ (Δ°C)		NS-EM (% NL^*a*^)	
				BG505	B41	BG505	B41
Adjuvant-free control				65.3	61.4	100	100
Alhydrogel	Aluminum hydroxide colloidal suspension	Alum	InvivoGen	+0.6	+2.0	100	100
Precipitated alum	Aluminum sulfate	Alum	EMD Millipore	−9.6/−2.2*	−8.9/+3.0*	15–40	60–70
GLA-LSQ	Liposome, QS21 saponin, GLA	Liposome	IDRI	+2.2	+0.5	100	100
MPLA liposomes	Monophosphoryl lipid A, liposome	Liposome	Polymun Scientific GmbH	−0.1	+0.2	100	100
ISCOMATRIX	QS21 saponin, cholesterol/DPPC in MEGA-10	ISCOM	CSL Ltd.	+0.2	+0.2	100	100
Quil-A saponin	Saponin	Amphipathic glycoside	InvivoGen	−0.6	−1.2	100	100
CpG (ODN 1826)	Synthetic oligodeoxynucleotide	Polyanion	InvivoGen	+1.9	−1.7	100	100
Poly(I·C)	Synthetic double-stranded RNA	Polyanion	InvivoGen	0	+0.2	100	100
Sigma Adjuvant System	MPLA and synthetic trehalose dicorynomycolate in 2% squalene-Tween 80-water	Oil-in-water emulsion	Sigma Aldrich	+0.4	+0.1	100	ND^*c*^

a NL, native-like.

b Top two peaks by intensity.

c ND, not determined.

**TABLE 3:** Adjuvant-trimer formulations

Adjuvant name	Adjuvant concn	
	EM/DSF^*a*^	ELISA^*b*^/BLI^*c*^
Adjuvant-free control	0	0
Alhydrogel	50%, vol/vol (5 mg/ml aluminum)	5%, vol/vol (0.5 mg/ml aluminum)
Precipitated Alum	5%, wt/vol (50 mg/ml aluminum)	ND^*d*^
GLA-LSQ	50%, vol/vol (10 μg/ml GLA)	5%, vol/vol (1 μg/ml GLA)
MPLA liposomes	10%, vol/vol (0.39 mg/ml)	1%, vol/vol (0.039 mg/ml)
ISCOMATRIX	375 U/ml	37.5 U/ml
Quil-A saponin	250 μg/ml	25 μg/ml
CpG (ODN 1826)	150 μg/ml	15 μg/ml
Poly(I·C)	10 μg/ml	1 μg/ml
Sigma Adjuvant System	50%, vol/vol (0.125 mg/ml MPLA)	5%, vol/vol (0.0125 mg/ml MPLA)

a Trimer concentration for all coformulations, 150 μg/ml.

b Trimers were added to ELISA plates at a fixed concentration of 1.5 μg/ml. The listed adjuvant concentrations were the highest tested; serial dilutions from this concentration were also assessed.

c Trimer concentration for all coformulations in the BLI format, 25 μg/ml.

d ND, not done.

### Alum-based adjuvants.

Aluminum salts (alum) are a frequently used adjuvant based on decades of safe use in human vaccines (27). There are many alum-antigen formulations, but we chose to study two representative methods: coprecipitation of trimers with aluminum sulfate (coprecipitation method) and simple adsorption of trimers onto an alum gel at a neutral pH (Alhydrogel method). While the two procedures have been generally considered to yield a similar result, i.e., a strong interaction between the antigen and alum, we found that they are not equivalent with respect to the quality of the trimers present in the final formulations.

**FIG 1:**
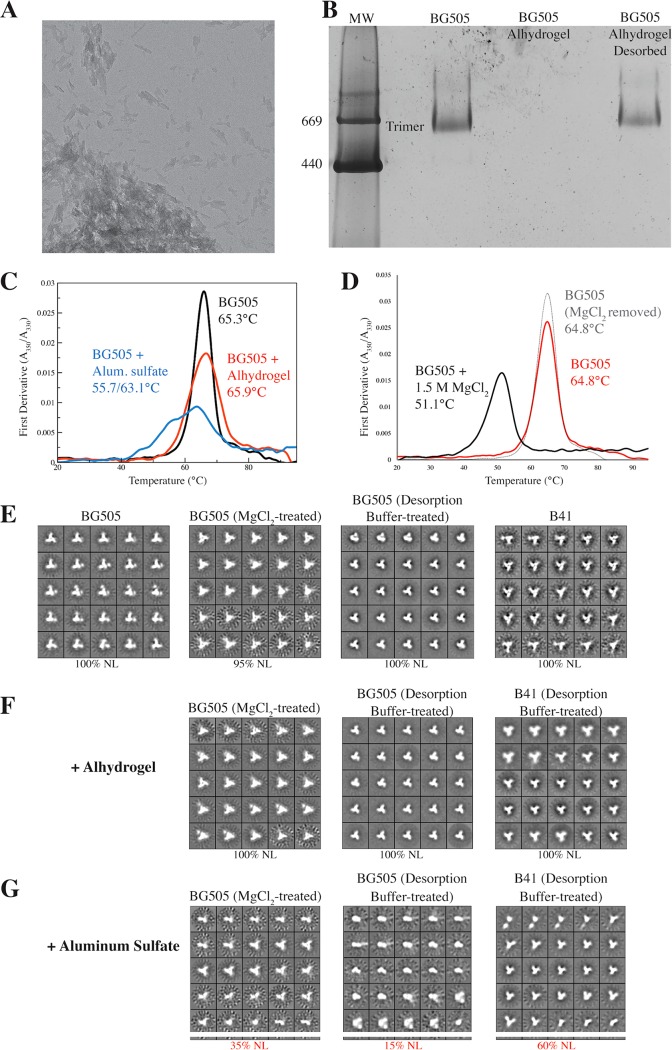
Alum adjuvant effects on trimer stability. (A) Representative electron micrograph of BG505 SOSIP.664 trimers adsorbed to Alhydrogel, with a field of view of approximately 840 nm by 840 nm. (B) BN-PAGE analysis of reference standard BG505 trimers, the same trimers adsorbed to Alhydrogel, and then desorbed using desorption buffer. The position of bands corresponding to trimers is indicated. MW, molecular weight, in thousands. (C) DSF analysis of how alum adjuvant affects the thermostability of BG505 trimers. The data were derived from formulations that did not undergo a prior desorption step. (D) DSF analysis showing that MgCl_2_ exposure destabilizes BG505 trimers but that the effect is reversible. The midpoint melting temperatures are recorded on each panel. (E to G) NS-EM analysis of reference standard BG505 or B41 trimers (as indicated) before and after exposure to buffer only (E), Alhydrogel (F), or aluminum sulfate (G), as indicated above each panel. The percentage of native-like (NL) trimers is shown under each panel.

In pilot studies, we found that alum adsorbs trimers so efficiently that we could not use NS-EM to directly image trimer-alum complexes. The micrographs were dominated by what appeared to be sheets of alum, with no adsorbed trimers visible (Fig. 1A). While sample dilution and disruption by ultrasonication each have been reported to facilitate NS-EM imaging of antigens adsorbed to Alhydrogel, the protein complexes used in those studies (i.e., keyhole limpet hemocyanin, Rous sarcoma virus n-RNA helices, and anthrax protective antigen PA63) were 3 to 100 times the mass of SOSIP trimers, which simplified the visualization process (10). We found that a similar approach was not helpful for visualizing alum-adsorbed SOSIP trimers, as their relatively small size and lack of asymmetric features prevented the stain from creating enough contrast between the trimers and the alum background. Blue native polyacrylamide gel electrophoresis (BN-PAGE) analyses of alum-trimer mixtures were also problematic, in that the alum particles interfered with the binding of the Coomassie blue stain to the trimers and either hindered their migration on the gel or otherwise prevented their visualization (Fig. 1B).

Unlike NS-EM and BN-PAGE, differential scanning fluorimetry (DSF) could be used for the direct analysis of adsorbed trimers. Adsorption to Alhydrogel resulted in modest and more substantial stabilization, respectively, of the BG505 (+0.6°C) and B41 (+2.0°C) trimers relative to the reference controls (Table 2 and Fig. 1C). Melting curves for trimers adsorbed to aluminum sulfate via the coprecipitation method were markedly different and displayed a high degree of heterogeneity. Thus, the BG505 trimers that were coprecipitated with alum melted over a wide temperature range, with initial and secondary peaks at 55.7°C and 63.1°C (Fig. 1C). The initial peak is at a temperature ∼6.8°C lower than the BG505 trimer reference standard thermal melting value of 65.3°C, implying that the coprecipitation formulation has driven some trimers to dissociate into dimers or monomers and/or partially denatured the individual protomers (Fig. 1C). The secondary peak, at a temperature 2.0°C lower than the reference standard, is an additional indicator of instability (Fig. 1C). After coprecipitation of the B41 trimers with alum, the corresponding peaks were at temperatures 8.4°C lower and 3.0°C higher than the reference standard value (61.4°C) (Table 2). The initial peak may again arise from trimer destabilization, while the secondary peak could reflect protein aggregation events.

Overall, we suggest that DSF is a useful initial screening tool for identifying problematic interactions between trimers and adjuvants due to its speed, its relatively low sample requirement, and its utility with antigen/adjuvant coformulations that are otherwise hard to analyze. For example, DSF is useful when the strength of the adjuvant-antigen interaction otherwise interferes with the analytical procedure, which we found could occur with BN-PAGE and NS-EM, or when adjuvant-derived particles create suspensions that are too opaque for NS-EM to be used.

To further probe the nature of the changes in stability caused by aluminum sulfate, we attempted to release trimers from preformed alum complexes and thereby allow them to be analyzed by NS-EM. We first tested a high-salt solution, based on our routine use of 3 M MgCl_2_ to elute BG505 SOSIP.664 trimers from antibody affinity columns without damaging their conformation (21, 28). The short-term exposure to a high (1.5 M) MgCl_2_ concentration transiently affected the thermal stability of the trimers, lowering their melting temperature by ∼14°C, but this effect was fully reversed once the salt was dialyzed out (Fig. 1D). NS-EM imaging confirmed that transient exposure to MgCl_2_ did not permanently damage the BG505 trimers (Fig. 1E). We therefore adsorbed BG505 trimers to alum using either the coprecipitation or Alhydrogel method and then attempted to desorb them using MgCl_2_ and a centrifugation-based process (see Materials and Methods). The outcome was that some BG505 trimers were successfully released from the alum particles and could be imaged by NS-EM (Fig. 1E to G). However, by measuring protein content (see Materials and Methods), we estimated that the MgCl_2_ method liberated only a small fraction (<5%) of the input trimers from the alum. It is, therefore, possible that the released subset is not representative of what remained alum associated.

An alternative approach using desorption buffer allowed a more efficient recovery of trimers, which we estimated to be ∼50 to 70% of the initial input (see Materials and Methods). While we cannot formally exclude the possibility that the recovered trimers differ biochemically or biophysically from what remains alum absorbed, we note that we do recover in excess of half the total population. It seems likely, therefore, that analyzing the recovered fraction does yield meaningful information. We found that exposure to desorption buffer alone did not cause visible damage to the trimers (Fig. 1E). NS-EM imaging further showed that BG505 or B41 trimers released from Alhydrogel using the desorption buffer method were 100% native-like with no indications of damage or degradation (Fig. 1F). In marked contrast, however, a significant fraction (30 to 85%) of the BG505 or B41 particles desorbed from the alum-trimer coprecipitation samples appeared to be monomeric or resembled other trimer degradation products (Fig. 1G and Table 2). The desorption buffer method also allowed BN-PAGE analyses to be performed; the BG505 Env proteins released from Alhydrogel migrated on the gel as a single band at a position corresponding to trimers (Fig. 1B).

Because of the strong interaction between the SOSIP trimers and alum particles, no single technique was sufficient to allow a definitive analysis. However, taken together, the above-described results raise serious concerns regarding the coprecipitation method. Thus, although some native-like SOSIP trimers can be recovered from the alum coprecipitates, we saw substantial evidence for the presence of degradation products. The coprecipitation method involves adding trimers in Tris-buffered saline (TBS; 20 mM Tris, 100 mM NaCl, pH 7.5) directly to an equal volume of an aluminum sulfate solution that is highly acidic (pH 2.6), creating a mixture with a pH that we measured as 2.9. Alkali is then gradually added to neutralize the mixture and promote the precipitation of alum particles containing absorbed trimers. A formulation of two parts antigen (trimers in TBS) to one part aluminum sulfate (2:1 ratio) had a pH of ∼3.0. Even at a 9:1 ratio, the pH remained acidic, at 3.6. Hence, a neutral-buffered saline solution is not sufficient to neutralize the acidity of aluminum sulfate at mixing ratios relevant to preparing immunization formulations. In contrast, mixing Alhydrogel (pH 7.4) with an equal volume of TBS (pH 7.5) resulted in only a modest change in pH, to 6.8. A prolonged (3-day) incubation of BG505 SOSIP.664 trimers in a pH 3.6 buffer is known to cause their complete disintegration into dimeric and monomeric components (21). It is reasonable to assume that even the much shorter exposure to acidic conditions that is inherent to the alum coprecipitation method would be problematic. In contrast, the Alhydrogel method involves absorbing the trimers onto alum particles at neutral pH; we saw no indications that this procedure was problematic. We conclude that alum association does not in itself detectably damage BG505 and B41 SOSIP trimers. However, exposing trimers to a highly acidic solution during the coprecipitation method seriously compromises their structural integrity and homogeneity. In summary, we have identified a sound rationale for using Alhydrogel but not the coprecipitation method as an alum-based adjuvant for SOSIP trimers.

### ISCOMs, saponins, and liposomes.

**FIG 2:**
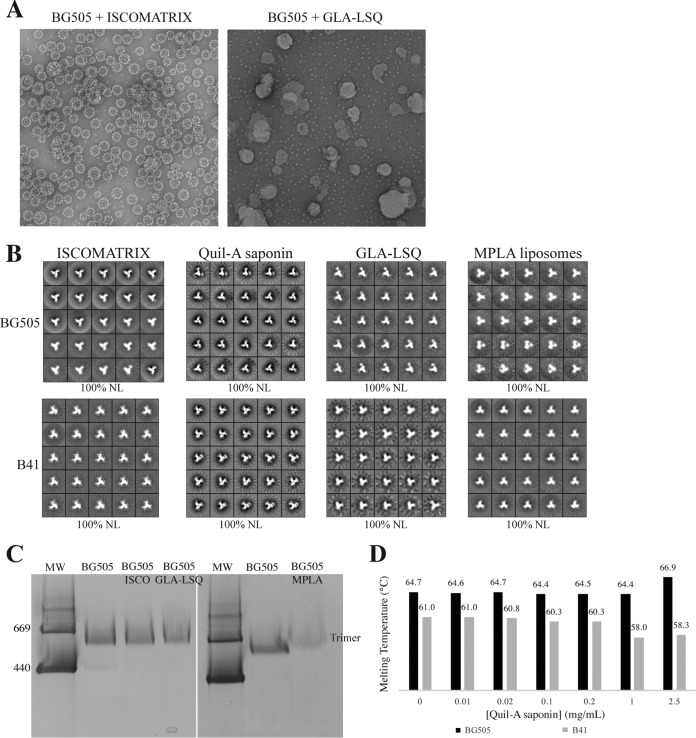
Trimer stability in the presence of ISCOMs, liposomes, and saponins. (A) Representative micrographs of BG505 trimers coformulated with ISCOMATRIX or GLA-LSQ, as indicated. (B) NS-EM analysis of BG505 or B41 trimers in the presence of ISCOMATRIX, Quil-A saponin, GLA-LSQ, or MPLA liposomes, as indicated. (C) BN-PAGE analysis of BG505 trimers coformulated with ISCOMATRIX (ISCO), GLA-LSQ, or MPLA liposomes (MPLA). The position of the bands corresponding to trimers is indicated. MW, molecular weight, in thousands. (D) The midpoint melting temperatures of BG505 or B41 trimers in the presence of the indicated concentrations of Quil-A saponin were measured by DSF.

An often-used adjuvant formulation involves mixtures of various lipids, surfactants, and/or saponins (20, 27, 29, 30). From an immunological standpoint, these various products can be further subdivided, but here we group them together because of one defining property: the adjuvant mixtures all form discrete nanometer- to micrometer-sized structures, often called ISCOMs, that are clearly distinguishable from BG505 SOSIP.664 trimer particles by NS-EM (5). As an example, we studied ISCOMATRIX, a product containing a purified mixture of saponins, including QS-21, along with cholesterol and phospholipids (20). We and others have successfully used this adjuvant in animal experiments (Table 1) (5, 7). NS-EM images of the mixture showed both the uniform, cage-like ISCOM particles and the much smaller trimers (Fig. 2A). We saw no indications that the trimers interacted directly with the ISCOMs, in that the two populations of particles appeared as clearly separate entities.

The clear separation of the components on the NS-EM grids allowed us to select and classify only the trimer particles; this analysis showed that the BG505 and B41 trimers remained 100% native-like in the presence of ISCOMATRIX (Fig. 2B). Furthermore, only a single band equivalent to the mass of a trimer was visible when a BN-PAGE gel was used to analyze the BG505 trimer plus ISCOMATRIX mixture (Fig. 2C). There was also no detectable change in the thermostability of either the BG505 or B41 trimers when ISCOMATRIX was tested by DSF (Table 2). Overall, we conclude that ISCOMs do not structurally compromise SOSIP trimers.

In a related set of experiments, we studied Quil-A, a less purified version of the QS-21 saponin component of ISCOMATRIX. Quil-A does not, by itself, form ISCOM-like cages, but it is sometimes used as an adjuvant in its own right (31, 32). Although DSF analyses suggested that both trimers were slightly destabilized (by −0.6°C for BG505 and −1.2°C for B41) when coformulated with Quil-A (Table 2), there was no evidence of visible damage in NS-EM images (Fig. 2B). The modest effect of Quil-A on the trimers in the DSF analysis was dose dependent; larger melting temperature changes of >2°C occurred at concentrations of free saponin in the 1 to 2.5 mg/ml range, which are likely to be much higher than those used in practice (e.g., 0.25 to 0.50 mg/ml in rabbits) (Fig. 2D and Table 3).

GLA-LSQ is a liposomal adjuvant containing glucopyranosyl lipid and QS-21 (33). As with ISCOMATRIX, the adjuvant components could be clearly distinguished from the BG505 and B41 trimers by NS-EM imaging of the mixture; the trimers were again not visibly associated with the liposomes, and they retained their fully native-like conformation (Fig. 2A and B). Consistent with the NS-EM images, GLA-LSQ affected neither the thermal stability of the trimers nor their migration as a single, trimer-sized band on a BN-PAGE gel (Table 2 and Fig. 2C). We also studied liposomes formed from bacterially derived monophosphoryl lipid A (MPLA) (34). These liposomes were, again, readily visible in NS-EM images, and they had no detectable effect on the coformulated BG505 or B41 trimers (see also data below on the Sigma Adjuvant System [SAS]) (Fig. 2B and C and Table 2).

### Oil-in-water emulsions and miscellaneous adjuvants.

**FIG 3:**
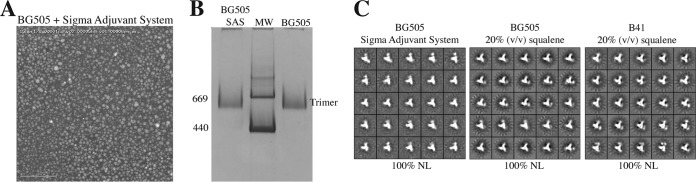
Trimers remain intact in the presence of oil-in-water adjuvants. Representative electron micrograph (A) and BN-PAGE gel (B) of BG505 trimers after coformulation with Sigma Adjuvant System (SAS). MW indicates molecular weight markers, in thousands, and a control BG505 trimer lane is also shown. (C) NS-EM analysis of BG505 or B41 trimers in the presence of SAS or 20% (vol/vol) squalene, as indicated.

Oil-in-water emulsions have long been used as adjuvants, exemplified by Freund's complete and incomplete adjuvants (12, 35). Updated formulations are commercially available, including the SAS product that we assessed as an example of this adjuvant class. The SAS, which supplanted the Ribi adjuvant system, contains MPLA and other immune-stimulating compounds in an emulsion formed by squalene, Tween 80, and water. In a pilot study, we found that the high background contrast caused by one or more SAS components, probably the oil droplets, made it difficult to image BG505 SOSIP.664 trimers by NS-EM (Fig. 3A). However, the thermal stability of either the BG505 or B41 trimers, as measured by DSF, was not affected by the presence of SAS (Table 2). In a BN-PAGE analysis, the dominant band was found at the position expected of a trimer (Fig. 3B). Because of concerns that oils or detergents may negatively impact the hydrophobic interactions between the gp140 protomers within an assembled trimer, we attempted to remove some of the sources of high contrast in NS-EM by a combination of centrifugation and chromatography (see Materials and Methods). This procedure allowed us to recover the trimer peak while reducing the background contrast sufficiently to allow NS-EM imaging. The resulting images showed that the recovered BG505 trimers were still intact and with a native-like conformation (Fig. 3C). In a further study, we found that one hydrophobic constituent, squalene, even when added at concentrations higher than are present in the SAS formulation, did not detectably affect the thermal stability of BG505 or B41 SOSIP trimers (Table 2). NS-EM images showed that BG505 or B41 trimers exposed to squalene retained their native-like conformation (Fig. 3C). It is likely that without additional surfactants the hydrophobic squalene molecules remain phase separated from soluble trimers. Finally, SAS is a suspension with a neutral pH of 7.0 to 7.5, assessed using litmus paper. We also found there was no measurable change in pH after mixing SAS or squalene with TBS to mimic the antigen formulation process. While no significant problems with the BG505 and B41 SOSIP trimers were identified using the conditions tested here, it is possible that various hydrophobic components of the SAS or other oil-in-water formulations adversely affect other genotypes of SOSIP trimers or less stable trimer designs. Appropriate analyses should be performed on a case-by-case basis.

### Polyanionic adjuvants.

**FIG 4:**
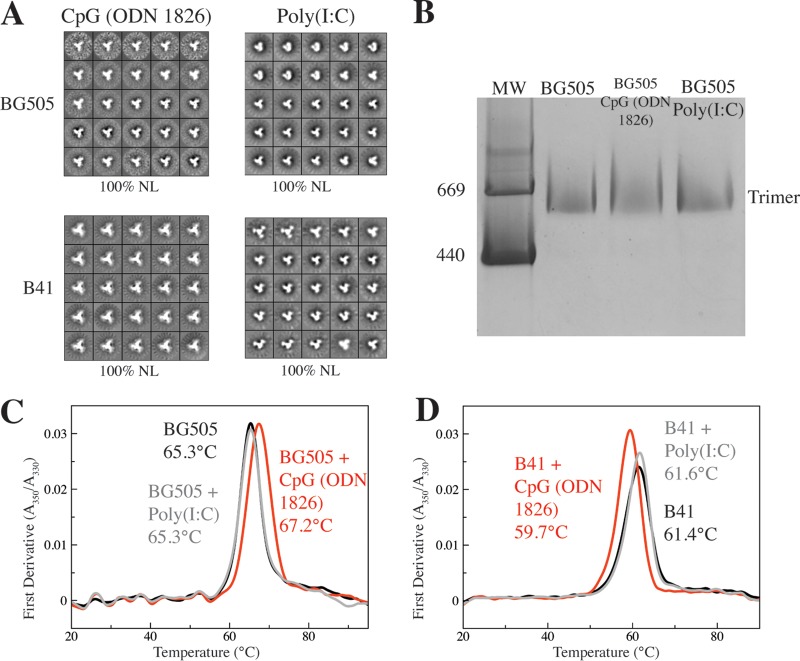
CpG (ODN 1826) but not poly(I·C) modulates the thermostability of BG505 and B41 trimers. NS-EM (A) and BN-PAGE (B) analyses of BG505 or B41 trimers after coformulation with either CpG (ODN 1826) or poly(I·C), as indicated. MW, molecular weight, in thousands. (C and D) DSF analyses show that CpG (ODN 1826) stabilizes the BG505 trimers but destabilizes B41 trimers, while poly(I·C) has no effect on the thermostability of either trimer. The midpoint melting temperatures are recorded on each panel.

Synthetic polynucleotides are polyanionic adjuvants based on TLR-activating pathogen-derived nucleic acids (36, 37). Here, we examined CpG (ODN 1826), a 20-mer DNA-based mimic of sequences commonly found in bacterial genomes, and poly(I·C), an analog of double-stranded RNAs that are early signals of viral infections. When mixed with the trimers in solution, neither polynucleotide formed structures large enough to be visualized by, or interfere with, NS-EM imaging. The resulting analyses showed that the BG505 and B41 trimers were fully native-like in the presence of either polynucleotide adjuvant (Fig. 4A). Similarly, BN-PAGE gels showed that BG505 trimers migrated as a single band of the expected size after mixing with either polynucleotide (Fig. 4B). However, CpG (ODN 1826) did alter the thermal stability of the trimers, by about +2.0°C for BG505 and −2.0°C for B41, whereas poly(I·C) had no effect on either trimer (Fig. 4C and D and Table 2). One interpretation of the DSF data is that CpG (ODN 1826) can bind to a SOSIP trimer, perhaps as a result of a charge-based interaction with cationic residues. Such an outcome could lead to the unwanted occlusion of a bNAb epitope(s), for example, ones involving the apex of the trimer where multiple electropositive amino acids are present (25). We describe below a wider-ranging study of how adjuvants, including the two polynucleotides, affect the antigenicity of the BG505 and B41 trimers.

### Effects of adjuvants on the antigenicity of SOSIP trimers.

**FIG 5:**
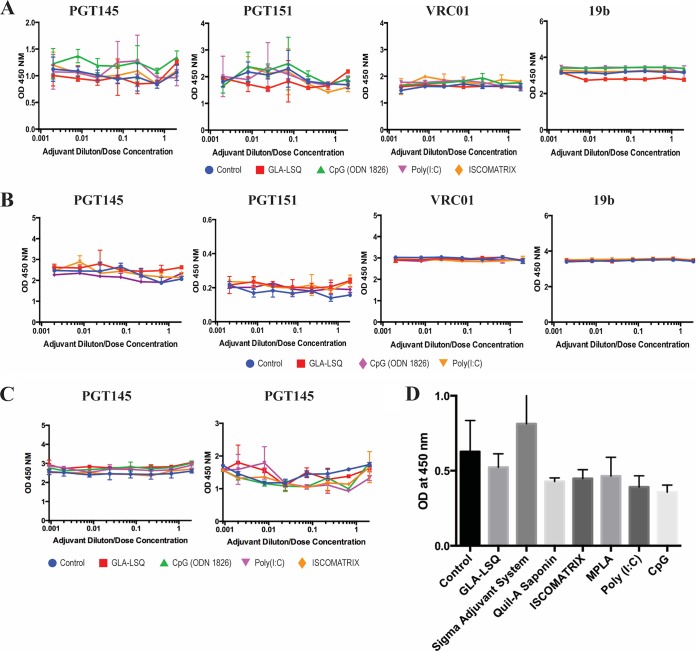
ELISA measurements of how adjuvants affect antibody binding to trimers. (A) The adjuvants indicated by the color scheme below the plots were added in a 50-μl volume at twice the indicated concentration to wells containing captured His-tagged BG505 trimers for a 4-h period at room temperature. The test antibodies (as indicated above each graph) were then added in a 50-μl volume for 4 h, the bound antibodies were detected, and the resulting OD_450_ value was recorded. (B) As for panel A, except that His-tagged B41 trimers were used. (C) As for panel A, except that the adjuvant-trimer incubation period was reduced to 30 min. The adjuvant was then either washed out (left) or not (right) before the addition of the PGT145 antibody and the completion of the assay. (D) 2G12-captured BG505 trimers were exposed to the indicated adjuvants for 1 h before PGT145 IgG was added for 1 h. The control group involved the addition of buffer only. Bound PGT145 was then detected and the resulting OD_450_ value recorded.

In light of the thermostability results described above, we first used a capture enzyme-linked immunosorbent assay (ELISA) to study whether the polyanions [CpG (ODN 1826) and poly(I·C)] and two adjuvants from another class (GLA-LSQ and ISCOMATRIX) affected the binding of three bNAbs and one non-NAb to various epitopes on C-terminally His-tagged versions of the BG505 SOSIP.664 and B41 SOSIP.v4.1 trimers (Fig. 5A and B). The bNAbs included a CD4 binding site epitope (VRC01), an epitope at the gp120-gp41 interface (PGT151), and one at the trimer apex (PGT145); we also used 19b, a non-NAb to a V3 epitope. The PGT145 and PGT151 epitopes are highly sensitive to trimer conformation, so any disruptive effects of adjuvants on the integrity of the trimers would be expected to reduce their binding; conversely, an increase in 19b binding could be an indicator of damage to the trimer structure that made this non-NAb epitope more accessible. Under the assay conditions shown, the trimers were first immobilized via their tags, and the adjuvants were added over a range of dilutions (with starting concentrations summarized in Table 3) for 4 h at room temperature. The test antibody was then included for a further 2 h before its trimer binding was quantified. None of the four adjuvants tested substantively modified the binding of any of the antibodies under these conditions (Fig. 5A and B). Of note is that CpG (ODN 1826), which did affect the thermostability of the trimers in DSF assays, had no detectable effect on any bNAb or non-NAb epitopes under the conditions of the His tag trimer ELISA. In additional experiments, we had found that neither washing away the adjuvants before addition of the test antibody nor using a shorter trimer-adjuvant incubation period of 30 min influenced the assay outcomes (Fig. 5C).

In a second ELISA-based approach, we captured nontagged trimers onto the solid phase via the adsorbed 2G12 bNAb (an IgG antibody directed against the gp120 high-mannose patch) and then added an adjuvant or adjuvant component (using the same concentrations tested in the His tag trimer ELISA; Table 3) for 1 h followed by the PGT145 bNAb to the trimer apex (Fig. 5D). We did not test precipitated alum in this assay because its acidity disrupted the integrity of trimers (described above). We also did not evaluate Alhydrogel, because pilot studies showed that residual alum particles caused a major increase in the binding of either PGT145 or the labeled anti-human IgG antibody to the solid phase, even after extensive washing procedures, leading to unacceptably high assay backgrounds (optical density at 450 nm [OD_450_] values of >3). None of the other adjuvants, including CpG (ODN1826), had a substantial effect on PGT145 binding under these assay conditions (Fig. 5D).

**FIG 6:**
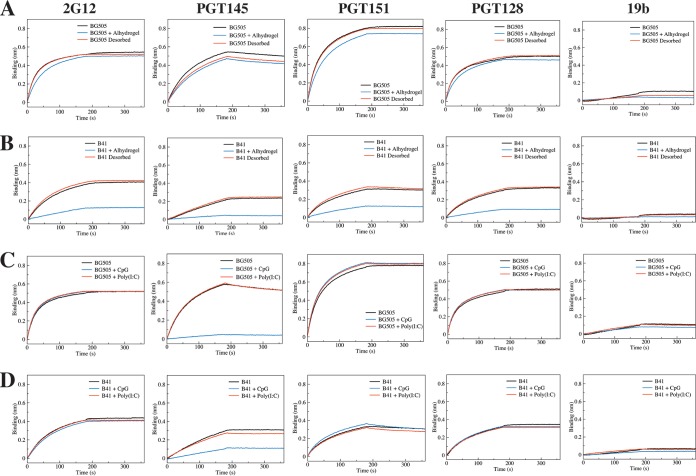
BLI measurements of how adjuvants affect antibody binding to trimers. The data shown represent the relative binding of 5 different IgGs to BG505 (A and C) or B41 (B and D) trimer, as indicated. (A and B) The trimers were absorbed to and then desorbed from Alhydrogel, as indicated. (C and D) The trimers were coformulated with CpG (ODN 1826) or poly(I·C), as indicated.

We next used biolayer interferometry **(**BLI) to further assess how Alhydrogel, CpG (ODN 1826), and poly(I·C) affected the antigenicity of nontagged BG505 or B41 trimers. In this study, we immobilized five IgG antibodies as antigenicity probes: bNAbs PGT145, PGT151, and 2G12, non-NAb 19b (see above for these epitopes), and bNAb PGT128 (V3-glycan epitope). In the BLI assay, Alhydrogel caused minimal to no interference in the binding of BG505 trimers to any of the immobilized antibodies (Fig. 6A). However, there was a variable but quite marked decrease in the binding of the B41 trimers to all four bNAbs, although without any increase in 19b non-NAb binding that might be indicative of an alum-induced opening of the trimer (Fig. 6B). The most likely explanation is that alum particles, even when diluted, are able to bind nonspecifically to trimers and decrease the access of certain antibodies to their epitopes. Variation in the distribution of charged residues across the surfaces of different trimers could account for why the B41 genotype appeared to be more affected than BG505. To further study this scenario, we treated the Alhydrogel formulations of BG505 or B41 trimers and adjuvant-free control trimers with desorption buffer (see Materials and Methods). In the subsequent BLI analysis, the PGT151, PGT128, 2G12, and 19b antibodies each bound comparably to the extracted adjuvant-exposed versus control trimer preparations, while PGT145 binding was again slightly decreased for the BG505 trimers but not for B41 (Fig. 6A and B). It is possible that, even after the extraction procedure, some residual alum particles still remain associated with the trimers in a way that modestly interferes with how the anionic PGT145 paratope interacts with the cationic apex of the trimer.

**FIG 7:**
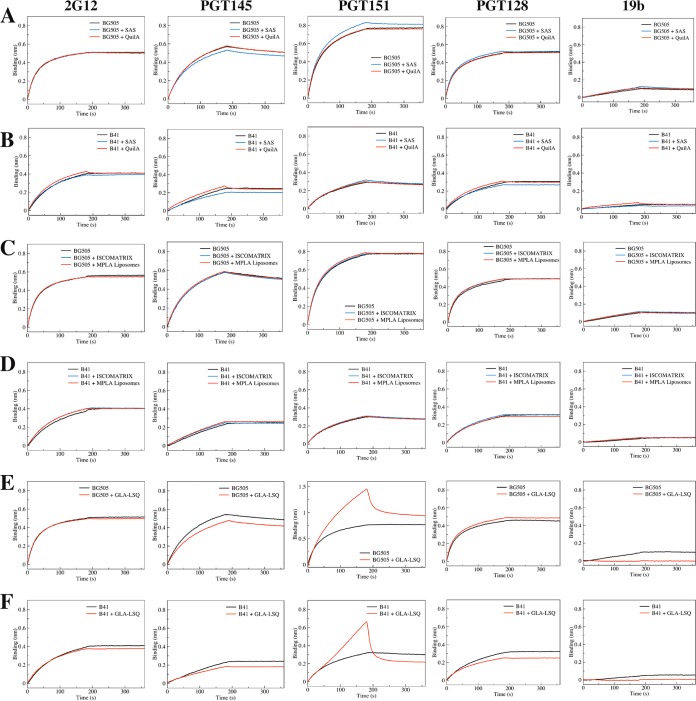
Additional BLI measurements of adjuvant effects on antibody binding to trimers. The layout is comparable to that for Fig. 6. The data shown represent the relative binding of 5 different IgGs to BG505 (A, C, and E) or B41 (B, D, and F) trimer, as indicated. The trimers were coformulated with SAS or Quil-A (A and B), ISCOMATRIX or MPLA liposomes (C and D), or GLA-LSQ (E and F).

The two polyanionic adjuvants were also tested in the BLI assay (Fig. 6; see also Fig. S1 in the supplemental material). A notable finding was that CpG (ODN 1826) completely blocked and substantially reduced PGT145 binding to the BG505 and B41 trimers, respectively (Fig. 6C and D). This outcome is consistent with the DSF data that implied there was a binding interaction between this adjuvant and the trimers (Fig. 4C and D). In contrast, the other polyanion, poly(I·C), had no effect on antibody binding to either trimer (Fig. 6C and D). The same finding was true of most of the other adjuvants, although GLA-LSQ caused a minor reduction in PGT145 binding to SOSIP trimers (note that we also observed that GLA-LSQ bound nonspecifically to the PGT151 antibody, which causes an artifactual reduction in PGT151-trimer association) (Fig. 7). The SAS product caused a small decrease in PGT145 binding and a correspondingly minor increase in PGT151 binding to the BG505 trimer (Fig. 7). It is possible that the latter finding arises because an unidentified hydrophobic SAS component increases the accessibility of the fusion peptide component of the PGT151 epitope. Whatever the reason, the SAS product has at most a marginal effect on the trimers, as no changes were detected by NS-EM or DSF (Fig. 3C and Table 2).

## DISCUSSION

Our goal was to gain an understanding of whether and how various types of adjuvants affect the stability, conformation, and antigenicity of this new generation of HIV-1 Env glycoprotein immunogens, such as SOSIP trimers. Adjuvants are of critical importance in vaccine development, and particularly so for HIV-1 Env proteins, which are notoriously poor immunogens (38). However, the chosen adjuvant must not have substantive adverse effects on the vaccine antigen, whether an HIV-1 Env trimer or the corresponding (glyco)protein from another pathogen. The true impact of an adjuvant can only be determined *in vivo*, but it is usually not possible to test every available formulation in animals or, even more so, in humans. Hence, an *in vitro* study can be valuable for identifying interactions between the adjuvant and the antigen that are sufficiently problematic to preclude more complex and expensive animal or human experiments. Here, we sought evidence for any adverse effects of various adjuvant formulations on the conformation of the BG505 SOSIP.664 and B41 SOSIP.v4.1 trimers, as judged by NS-EM, on their stability and integrity, assessed by BN-PAGE and DSF, and on their antigenicity for selected bNAbs, determined by ELISA and BLI.

We first assessed whether the various adjuvants damaged the conformational integrity of the SOSIP trimers. The main conclusion is that alum adjuvant formulations involving coprecipitation of trimers with aluminum sulfate should be avoided; the exposure to low pH inherent in this procedure seriously damages trimers by causing them to dissociate into monomers and/or other nonnative forms. The concerns about the acidic pH should apply to other immunogens, Env related or not, formulated in aluminum sulfate, particularly if the antigen is multimeric or otherwise conformationally or pH sensitive. In contrast, the neutral pH Alhydrogel formulation had no discernible effect on SOSIP trimer integrity and seems entirely appropriate for future studies *in vivo*. The same conclusion is likely to apply to other alum formulations prepared at neutral pH, such as Adju-Phos (39).

The ISCOM class was represented in our study by ISCOMATRIX, an adjuvant that we had used successfully in animal studies that were preceded by *in vitro* analyses showing it had no adverse effect on trimer conformation or antigenicity (5, 23, 40). We confirmed and extended those findings and also observed that the trimers did not bind detectably to the ISCOM cages. The basis of the ISCOM concept is the formation of lipid plus detergent cages of a size similar to that of common viral pathogens, with the vaccine antigens adhering to the surface of the cages via predominantly hydrophobic interactions or intercalating within their hollow interior (20, 29, 30, 41). The presentation of antigens in particulate form is known to confer immunogenicity advantages (42–44). As noted, we saw no evidence that the SOSIP trimers, which lack a membrane-interactive domain, became associated with the ISCOM cages, although it is possible that some trimers were present within the cages but were invisible. How ISCOMATRIX and other members of this adjuvant class, such as Matrix-M, successfully boost the immunogenicity of HIV-1 Env proteins, including SOSIP trimers, is likely to be rooted in other, immunology-based explanations (5, 20, 23, 29, 30, 40, 45, 46). The liposomes present in the GLA-LSQ and MPLA formulations also did not detectably associate with SOSIP trimers, and these adjuvants also had no adverse effect on trimer integrity, stability, or conformation. Taken together, we identified no reason to not evaluate these adjuvants, and presumably others of broadly similar designs, in clinical studies.

The commercially available SAS product served as a representative of the adjuvant class based on oil-in-water emulsions that include detergents/surfactants. In BLI studies, we found some indications that SAS modestly perturbs the structure of SOSIP trimers, although not to the extent of causing them to dissociate into dimers or monomers. One SAS component, squalene, had no measurable impact on trimer stability when tested alone. Future studies should address whether squalene remains as inert when formulated with a surfactant (e.g., in the MF59 adjuvant formulation). It would be prudent to investigate specific antigen-adjuvant mixtures in detail before selecting an oil-in-water emulsion. We note that, 20 years ago, the oil-in-water-based complete Freund's adjuvant was shown, or at least strongly suspected, to have damaging effects on the conformation of monomeric gp120 proteins, which of course lack the quaternary structural properties of trimers (12).

Based on *a priori* considerations of charge-based associations and an awareness of reports that they inhibit HIV-1 entry *in vitro*, we suspected that the highly polyanionic polynucleotides poly(I·C) and CpG (ODN 1826) interact directly with cationic regions of the trimer (13–19). We found indications that this could, in fact, take place. Most notably, CpG (ODN 1826) increased (for BG505) or decreased (for B41) the thermal stability of SOSIP trimers by ∼2°C, a change comparable to what can be achieved by structure-guided sequence modifications to the same trimers (40, 46). These effects were not seen with poly(I·C), which, although RNA and not DNA based, should still present a strong negatively charged phosphate backbone. The length and three-dimensional arrangement of anionic polymers may determine the extent to which they bind to SOSIP trimers (see below).

The cationic trimer apex region is a plausible binding site for a polyanionic adjuvant, such as CpG (ODN 1826) or poly(I·C), and contains key bNAb epitopes exemplified by the PGT145 site (25, 47). The apex is also a conformationally flexible region of the BG505 trimer and, to a greater extent, its B41 counterpart (26, 48). In the BLI assay, CpG (ODN 1826) strongly occluded the epitope for PGT145 but not those for other bNAbs, whereas poly(I·C) did not inhibit the trimer binding of any of the bNAbs. None of the other adjuvants substantively affected trimer antigenicity in the BLI assay after we controlled for background and nonspecific binding of adjuvant components to the biosensors or chips (see Materials and Methods). In contrast to BLI, CpG (ODN 1826) did not affect PGT145 binding in the His-tagged trimer ELISA. We had expected that this method would be a flexible way to obtain a baseline data set on how adjuvants affected a variety of bNAb epitopes. The inability of the His tag ELISA to detect the PGT145 epitope-occluding effect of CpG (ODN 1826) is, however, a concern. It is well established that capture ELISAs using C-terminally tagged SOSIP trimers falsely report on the antigenicity of the V3 region by indicating that it is well exposed for non-NAb binding when other techniques clearly show otherwise (6, 49). Similar to the V3 epitope, the PGT145 epitope and the likely CpG (ODN 1826) binding site are all located near the trimer apex; capturing SOSIP trimers to solid phases via C-terminal tags must somehow affect the local conformation of this region. However, we also saw no inhibition of PGT145 binding by CpG (ODN 1826) in an alternative ELISA format, where nontagged trimers were captured via adsorbed 2G12. Overall, we recommend the use of BLI over ELISA for probing how adjuvant-trimer interactions affect bNAb and non-NAb epitopes.

The methods that we describe here could be used, or adapted, to study additional adjuvant concepts that are now being developed, and they could also be adopted for various immunogens of medical interest. The composition of the adjuvant and the nature of the immunogen will dictate what methods are most suitable and how they may need to be modified. For example, HIV-1 Env proteins are being presented on the surfaces of liposomes or protein-only nanocages (50, 51). The physical integrity of the particles and their retention of the Env protein could be sensitive to various adjuvant components and should be assessed on a case-by-case basis.

Ultimately, the benefits conferred by an adjuvant can only be definitively determined *in vivo*. However, the approach we have described here could identify adjuvants that are unsuitable for *in vivo* testing with a particular immunogen, and thereby reduce, at least to a degree, the cost and complexity of vaccine development programs.

## MATERIALS AND METHODS

### Trimer production and purification.

The BG505 SOSIP.664 and B41 SOSIP.v4.1 trimers were produced in stable CHO cell lines and purified by 2G12 bNAb affinity chromatography followed by size exclusion chromatography (SEC), as described previously (26, 28). C-terminally His-tagged versions of the same trimers were used only in ELISAs; they were produced by transient transfection of FreeStyle HEK293F cells (Thermo Fisher) and purified by PGT145 bNAb affinity chromatography followed by SEC (26, 49). The trimer concentration was adjusted to 0.5 mg/ml in Tris-buffered saline (TBS; 20 mM Tris, 100 mM NaCl, pH 7.5), aliquoted, and frozen.

### Sources of adjuvants.

Aluminum sulfate was purchased from EMD Millipore (AX0735-1; Billerica, MA) and dissolved in distilled water to create a 10% (wt/vol) stock solution. Alhydrogel (vac-alu-250), CpG (ODN 1826) (tlrl-1826-1), poly(I·C) (tlrl-pic; high molecular weight), and Quil-A (vac-quil) were all purchased from InvivoGen (San Diego, CA). The Sigma Adjuvant System (S6322) and squalene (S3626) were purchased from Sigma-Aldrich (St. Louis, MO). The former is compositionally similar to, and has replaced, the Ribi adjuvant system. ISCOMATRIX produced by CSL Ltd. (Melbourne, Australia) was obtained from the International AIDS Vaccine Initiative (IAVI). GLA-LSQ, a glucopyranosyl lipid adjuvant liposome containing the saponin QS21, was provided by the Infectious Disease Research Institute (Seattle, WA) via the Bill and Melinda Gates Foundation's Collaboration for AIDS Vaccine Discovery program. MPLA liposomes were a gift from Polymun Scientific Immunbiologische Forschung GmbH (Klosterneuburg, Austria).

### Formulation and incubation of adjuvant-trimer mixtures.

For Alhydrogel, GLA-LSQ, and the SAS, 30 μg of trimers (100 μl of a 0.3-mg/ml stock) was mixed with 100 μl of the adjuvant stock solution (e.g., the commercial product) followed by incubation for 1 h at room temperature. For other adjuvants, sufficient TBS, pH 7.5, was added to bring the final volume up to 200 μl. Specifically, the trimers were mixed with ISCOMATRIX (75 U), CpG (ODN 1826) (30 μg, i.e., 15 μl of a 2-mg/ml solution), poly(I·C) (2 μg, i.e., 2 μl of a 1-mg/ml solution), MPLA liposomes (20 μl), or Quil-A (50 μg, i.e., 5 μl of a 10-mg/ml solution). Squalene was tested at concentrations of up to 20% (vol/vol). For a summary of formulations, see Table 3.

The procedure used to test precipitated aluminum sulfate was as follows. After mixing the trimers with an equal volume (100 μl) of 10% aluminum sulfate solution (dissolved in water and sterile filtered; EMD Millipore), a solution of 1 M KOH was added drop by drop until the pH reached ∼6.5 (as determined with litmus paper). TBS was added up to 2 ml, and the precipitated complex was centrifuged using a tabletop centrifuge at 600 × *g* for 10 min. The supernatant was discarded and the pellet was washed 3 times by resuspension in TBS and centrifugation. After the final wash, the pellet was resuspended in 200 μl of TBS.

### Desorption of trimers from alum particles.

Alum-formulated trimer samples were diluted either 1:2 with 3 M MgCl_2_ for 15 min or 1:3 with desorption buffer (10% [wt/vol] sodium citrate, 1 M NaCl, 0.025 M sodium phosphate, pH 6.8) for 60 min. After incubation in either buffer, the alum particles were pelleted by centrifugation for 10 min at 10,000 × *g*, and the soluble fraction was concentrated and buffer exchanged into TBS. To control for any adverse effects of exposure to MgCl_2_ or desorption buffer, adjuvant-free trimers were also subjected to the above-described procedures, although without the centrifugation step. The extent of sample loss during the concentration and buffer exchange steps was assessed by measuring the total protein content of both adjuvant-free (control) and alum-desorbed trimers via absorbance at 280 nm (UV_280_). The percent recovery of trimers from the alum formulations was then calculated from the two measurements.

### Negative-stain electron microscopy.

In most cases, the above-described trimer-adjuvant mixtures were diluted in TBS to a final trimer concentration of ∼0.02 to 0.03 mg/ml prior to NS-EM imaging and analysis as previously described (26, 46). The development of new procedures required for NS-EM analysis of trimer-alum complexes is described above. Following the 1-h incubation, trimer-SAS mixtures were centrifuged for 10 min at maximum speed using a tabletop microcentrifuge to separate the immiscible layers in the mixture of SAS with BG505 trimers, and then the bottom, aqueous phase was processed through a Superose 6 Increase (GE Healthcare) SEC column to complete the separation of the trimers from the adjuvant components. The pooled SEC fractions were then diluted and stained as described above.

### Blue native polyacrylamide gel electrophoresis.

The above-described standard conditions for the formulation of adjuvant-trimer mixtures were used, and the samples were incubated for 1 h at 37°C. An aliquot of each incubation mixture that contained 2 μg of the trimers was then mixed with loading dye and applied to a 4 to 16% Bis-Tris native PAGE gel (Invitrogen). The gels were run for 1.5 h at 200 V (0.07 A) using 50 mM Tris, pH 7.7, as the running buffer (Invitrogen) and then stained with SimplyBlue SafeStain (Invitrogen) (6).

### Differential scanning fluorimetry.

Trimer-adjuvant mixtures (described above) were loaded into glass capillary tubes for a thermal denaturation scan using a Prometheus NT.48 NanoDSF instrument (NanoTemper Technologies) with a heating ramp of 1°C/min. The instrument software determined the thermal transition points automatically. As controls, capillary tubes containing only the adjuvants at the final formulation concentrations were also processed to assess whether their components caused fluorescence changes at the wavelengths (330 and 350 nm) recorded by the instrument. No background interference effects were observed using any of the tested adjuvants.

### Biolayer interferometry.

The trimers were formulated as described above (formulation and incubation of adjuvant-trimer mixtures), except that the starting protein concentration was increased to 0.5 mg/ml and phosphate-buffered saline (PBS) was used in place of TBS. These alum-trimer suspensions were diluted 1:10 in kinetics buffer (PBS, pH 7.2, supplemented with 0.01% [wt/vol] bovine serum albumin and 0.002% [vol/vol] Tween 20). Alternatively, trimers were separated from Alhydrogel using desorption buffer (described above for desorption of trimers from alum particles). In this case, control (alum-free) trimers were also mixed with desorption buffer prior to buffer exchange into PBS and then used as reference standards for the BLI measurements. Test antibodies, as IgGs, were loaded onto anti-human IgG Fc capture (AHC) biosensors (ForteBio) and dipped into the diluted alum-trimer samples (estimated final trimer concentration, ∼110 nM) or alum-desorbed trimers (estimated final trimer concentration, ∼110 nM) using an Octet Red96 instrument (ForteBio). Trimer-antibody association was measured for 180 s, followed by dissociation for 180 s in kinetics buffer. For measuring nonspecific binding to the test antibodies, reference wells contained kinetics buffer with or without adjuvant at the same final concentrations as the trimer-alum samples. The resulting background curves were subtracted from each experimental data set (e.g., background values for Alhydrogel alone were subtracted from the Alhydrogel-trimer curves, and background values for kinetics buffer-alone data were subtracted from trimer-alone data). The resulting antibody-trimer binding curves were then processed by first aligning them on the *y* axis using the baseline step immediately preceding the association phase and then applying an interstep correction between the association and the dissociation curves. All data-processing steps were performed using the ForteBio data-processing software included with the instrument.

### ELISA using His-tagged trimers.

The ELISA was based on BG505 SOSIP.664 or B41 SOSIP.v4.1 trimers with a C-terminal His tag, the engineering and purification of which have been previously described (49). The trimers (100 μl of a 1.5-μg/ml solution in TBS) were captured overnight onto Ni^2+^-coated wells of a nickel-nitrilotriacetic acid (Ni-NTA) ELISA plate (Qiagen). After washing away unbound trimers, the wells were blocked for 30 min with a solution of 2% (wt/vol) skimmed milk in TBS. The test adjuvant, diluted in 50 μl of PBS, was added as a series of serial dilutions (with the highest adjuvant concentration listed in Table 3) for a variable time at room temperature. The test antibody was diluted in PBS plus 20% sheep serum and 2% skimmed milk powder and added to the adjuvant-containing wells at a predetermined concentration in a volume of 50 μl for a further 2 h. Alternatively, the adjuvant was removed by washing the wells three times with TBS before adding the test antibody (100 μl) for a further 2 h at room temperature. In both versions of the assay, the wells were then washed three times with TBS and the bound antibody was detected using a horseradish peroxidase-conjugated goat anti-human IgG (Bio-Rad) followed by the 1-step Ultra TMB-ELISA substrate color development solution (Pierce). The optical density at 450 nm (OD_450_) was measured after color development was terminated by the addition of sulfuric acid.

### 2G12 capture ELISA.

The 2G12 bNAb was coated onto wells of a Nunc Maxisorp ELISA plate (VWR) by overnight incubation at 1 μg/ml in 200 mM sodium carbonate-bicarbonate buffer, pH 9.4. The wells were blocked with 2% (wt/vol) skimmed milk and 10% goat serum in TBS for 1 h at room temperature. Nontagged BG505 SOSIP.664 or B41 SOSIP.v4.1 trimers (100 μl of a 1.5-μg/ml solution in PBS) were then captured onto the absorbed 2G12 antibody by incubation for 1 h at room temperature. The remaining procedures for addition of adjuvants, detection monoclonal antibody, and color development were as described above (ELISA using His-tagged trimers), except that the detection MAbs were biotin labeled and were detected with a streptavidin-conjugate of horseradish peroxidase (Pierce).
